# Development and validation of an inflammatory bowel disease nutrition self-screening tool (IBD-NST) for digital use

**DOI:** 10.3389/fnut.2023.1065592

**Published:** 2023-02-03

**Authors:** Catherine L. Wall, Bridgette Wilson, Miranda C. E. Lomer

**Affiliations:** ^1^Department of Nutritional Sciences, King’s College London, London, United Kingdom; ^2^Department of Medicine, University of Otago, Christchurch, New Zealand; ^3^Guy’s and St Thomas’ NHS Foundation Trust, London, United Kingdom

**Keywords:** inflammatory bowel disease, nutrition screening tool, dietitian, malnutrition, self-management, e-health, subjective global assessment

## Abstract

**Background and aim:**

The identification of, and timely intervention for, patients with impaired nutritional status may reduce inflammatory bowel disease (IBD) complications. This study aimed to develop and validate an IBD-specific nutrition self-screening tool (IBD-NST) that identifies patients at nutrition risk.

**Methods:**

An expert IBD panel was consulted to support development of an IBD-NST. The tool was assessed in different cohorts of patients attending IBD outpatient clinics for face, content and convergent validity and repeat reliability. The tool was compared with (i) the malnutrition universal screening tool to assess face validity and (ii) subjective global assessment (SGA), hand-grip strength (HGS) and mid-arm muscle circumference to assess convergent validity. Tool content was informed by agreement between assessment tools, sensitivity analysis and chi-squared tests. The IBD-NST was completed electronically twice, 1 week apart to assess repeat reliability using observed agreement and kappa statistic. Statistical significance assumed at *p* < 0.05.

**Results:**

In total, 282 IBD patients (175 with Crohn’s disease) were recruited to validate the IBD-NST. The final validated IBD-NST includes body mass index (BMI), weight loss and IBD-specific nutrition-focussed questions which were acceptable to patients. It identified patients at risk of malnutrition, moderately or severely malnourished patients and patients at nutritional risk. The IBD-NST identified 54/179 (30%) patients at moderate or high nutrition risk and had excellent repeat reliability in 85 patients [*r* = 0.77 (95% CI 0.669 to 0.746)].

**Conclusion:**

The IBD-NST is a self-screening tool, validated for use as either a paper or e-health version, that identifies patients at nutrition risk who are likely to benefit from dietetic assessment and intervention. Furthermore, patients with IBD symptoms who are concerned about their dietary intake can potentially access dietetic care more easily therefore encouraging greater self-management of IBD-related symptoms. The routine use of the IBD-NST as a self-screening tool would enable patient-led care in the outpatient setting and may facilitate timely access to dietetic care.

## 1. Introduction

Inflammatory bowel disease (IBD) is a chronic relapsing and remitting condition that has a profound negative impact on the gastrointestinal tract and, consequently, dietary intake, leading to an increased risk of malnutrition (1). The most commonly reported marker of poor nutritional status is low body mass index (BMI) (2). Historically, low BMI was relatively common in patients with IBD, however, average BMI of many healthy and IBD populations has increased over time (3, 4). An increase in BMI in the IBD population does not necessarily correspond to improved nutritional status (1). BMI is a reliable predictor of fat mass but not lean body mass or muscle function (5) which, are often lower in IBD cohorts compared with healthy populations (2). A BMI less than 18.5 kg/m^2^ is associated with increased risk of malnutrition (6) but in IBD hand-grip strength (HGS) and mid arm muscle circumference are more reliable anthropometric assessments of impaired nutritional status in patients without low BMI (5, 7). Reduced muscle mass and strength are associated with perceived fatigue and reduced quality of life (8–10). Furthermore impaired food-related quality of life is associated with increased disease activity, and from a nutritional perspective, reduced dietary fibre intake, and lower intake of nutrients important for bone health (11, 12). The identification of patients with IBD who have impaired nutritional status, and are not malnourished, may enable more timely nutrition intervention and thus improve patient nutritional status and ultimately quality of life (13).

Functional symptoms are common during quiescent IBD (14), negatively affect patient quality of life (15) and are challenging for both patients and clinicians to determine the symptom origin (14). Regardless of the symptom pathology, anecdotally patients often describe any IBD-related symptoms as a “flare” whether they arise from inflammatory, fibrotic or functional causes. Referral to an IBD dietitian is one option to address the burden of functional symptoms. Diet and lifestyle modifications are recommended (14) and effective (16) treatments in patients with IBD and should be delivered by dietitians to ensure nutritional adequacy of the diet and not further increase risk of impaired nutritional status (14). Current nutrition screening tools used in IBD practice tend to focus more on acute disease (17) and therefore unlikely to identify this group of patients who may benefit from a dietitian consultation.

A variety of nutrition screening tools have been used in IBD populations (17) and one IBD specific nutrition screening tool is available (18). This tool has good sensitivity for measuring nutrition risk in patients with a BMI > 25.0 kg/m^2^, but not specificity. It does not identify patients who are malnourished according to the Global Leadership Initiative on Malnutrition (GLIM) criteria (19) and further validation is needed.

The aim of this research was to develop and validate an IBD-specific nutrition self-screening tool (NST) for use with an IBD outpatient population that can identify patients at nutrition risk who are most likely to benefit from consulting with an IBD dietitian.

## 2. Materials and methods

### 2.1. Study design

**FIGURE 1:**
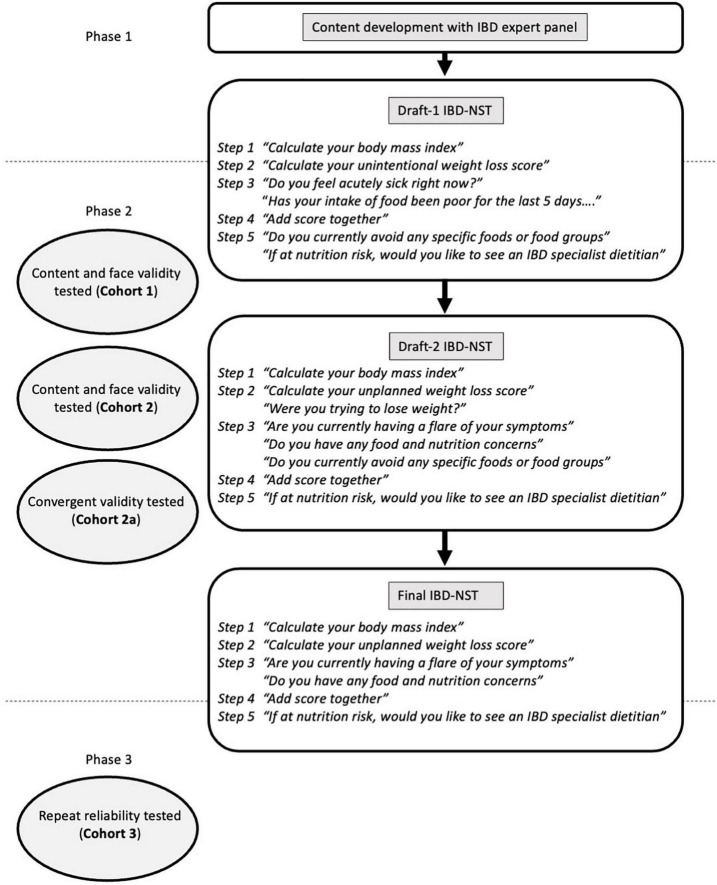
Development and validation of an IBD nutrition self-screening tool (IBD-NST).

The IBD-specific nutrition self-screening tool (IBD-NST) was developed in three phases: content development (phase 1); face, content and convergent validity (phase 2); and repeat reliability (phase 3) (Figure 1). Different cohorts of patients with IBD were recruited to test the face, content and convergent validity and repeat reliability of the IBD-NST. Patients with IBD were recruited from gastroenterology outpatient clinics at Guy’s and St Thomas’ NHS Foundation Trust, London, United Kingdom between 2018 and 2020. The inclusion criteria were patients with a documented diagnosis of IBD aged at least 16 years old and able to provide written informed consent. The only exclusion criterion was inability to read and understand English. All patients provided written informed consent.

Ethical approval was provided by North West–Liverpool East Research Ethics Committee (reference 18/NW/0062) and South Central–Oxford C Research Ethics Committee (19/SC/0479) and was approved by the NHS Health Research Authority. The research was conducted in accordance with the Declaration of Helsinki (1996).

### 2.2. Measurement tools

All patients completed a demographic and IBD history questionnaire. Patients reported weight and height in metric (kilogrammes and metres), and/or imperial (stones/pounds and feet/inches) measurements, respectively. Where patients did not know their height, an outpatient clinic stadiometer was used to measure their height (without shoes) in cm to the nearest 0.5 cm. Where patients did not know their weight, electronic outpatient clinic scales were used to measure their weight (without shoes) in kg to the nearest 0.1 kg. Weight, height, and BMI conversion charts were available to enable patients to complete the MUST and IBD-NST questions related to these measurements.

#### 2.2.1. Development of the IBD specific nutrition self-screening tool (IBD-NST)

Questions for the first version of the IBD-NST (Draft-1 IBD-NST) were developed by the authors in consultation with an expert IBD panel which included two patients with IBD, two IBD dietitians, one IBD nurse specialist, three gastroenterologists, one colorectal surgeon and a representative from Crohn’s and Colitis UK. MUST was used as a starting point to develop an IBD-specific tool alongside two novel and subjective questions (Figure 1). MUST is a validated malnutrition risk screening tool that uses a scoring system whereby the score is categorised as low (score 0), moderate (score 1) or high (score ≥2) risk of malnutrition based on BMI, unintentional weight loss, and acute disease effect (20).

#### 2.2.2. Face validity

The face validity of all versions of the IBD-NST were assessed qualitatively by a researcher. Patients recruited to Cohort 1 independently completed Draft-1 IBD-NST in the presence of a researcher in a dedicated clinic room. The Draft-1 IBD-NST included the MUST and two novel IBD questions (Figure 1). Once patients had completed all questions, they independently calculated their MUST score. While patients completed the Draft-1 IBD-NST, the researcher listened to, and noted, any patient comments and informal feedback, for example if patients asked for clarification regarding the meaning of a question or how to interpret the conversion charts to calculate BMI or percentage weight loss. The informal qualitative patient feedback from Cohort 1 and the expert IBD panel informed changes to the layout and score calculation components of the Draft-1 IBD-NST resulting in development of the Draft-2 IBD-NST. Similarly, informal qualitative patient feedback from Cohort 2 informed the development of the paper and electronic final IBD-NST.

Cohort 2 and Cohort 3 only completed questions relating to BMI and weight loss in the IBD-NST, and not the MUST, to avoid confusion at being asked similar questions twice. For this reason, it was not possible to test the face validity of the IBS-NST unplanned weight loss question compared with the MUST unintentional weight loss question.

#### 2.2.3. Content validity

The content validity of the Draft-1 IBD-NST novel questions were explored with Cohort 1. It was theorised that patients with food and nutrition concerns would not necessarily be identified as at risk of malnutrition on the MUST but would be more likely to want to see an IBD dietitian to discuss their food and nutrition concerns. The responses from Cohort 1 informed the content of Draft-2 IBD-NST.

The content validity of the Draft-2 IBD-NST, compared with the MUST, was tested with Cohort 2.

Cohort 3 patients tested the content validity of the Final IBD-NST. Cohort 3 completed an electronic version of the final-IBD-NST and MUST in a dedicated clinic room in the presence of a researcher and 1 week later at home from a link in an email. The electronic version calculated BMI and percentage weight loss score based on the data entered by patients. Patients were required to interpret the BMI and weight loss score to calculate an IBD-NST nutrition risk score.

#### 2.2.4. Convergent validity

To test convergent validity of the Draft-2 IBD-NST a subset of Cohort 2 (Cohort 2a) was invited to have a subjective global assessment (SGA) to assess for the presence or absence of malnutrition.

The SGA is a validated nutrition assessment tool (21) widely integrated into usual clinical dietetic practice. It comprises a clinical assessment (weight history, dietary intake, gastrointestinal symptoms, and functional capacity) and a physical assessment of fat and muscle stores (21). The SGA can be scored as one of three ratings: SGA-A well-nourished; SGA-B mild to moderately malnourished; SGA-C severely malnourished. Additional measurements of muscle function and muscle mass were conducted as surrogate markers of impaired nutritional status.

Muscle function was assessed by HGS measured in triplicate (Takei 5401 hand grip dynamometer, Niigata City, Japan) in a seated position with the non-dominant arm at 90° and exerting maximum force (2). Impaired muscle function (suboptimal HGS) was defined as a maximum HGS from three attempts that was less than 85% of the mean for age and sex (22).

Muscle mass was estimated by one researcher to prevent inter-rate bias using mid-upper arm muscle circumference (MAMC). This was calculated from mid-upper arm circumference and tricep skinfold.


(mid-upperarmcircumference-(tricepskinfoldx(3.1416/10))

Mid-upper arm circumference was measured in triplicate using a flexible tape measure at the mid-point between the acromion on the shoulder and the olecranon process at the elbow (2). At the mid-point, tricep skinfold was measured in triplicate (Harpenden skinfold calliper, Baty International, Burgess Hill, West Sussex, England). Depleted muscle mass was defined as a MAMC less than 5th centile for age and sex (23).

#### 2.2.5. Repeat reliability

The repeat reliability of the final electronic IBD-NST was assessed with patients in Cohort 3. The software programme Qualtrics (Qualtrics, Provo, UT, USA) was used to develop the electronic IBD-NST questionnaire. The electronic IBD-NST enabled patients to enter their weight and height in metric (kilogrammes and metres), imperial (stones and pounds and feet and inches) or a mixture of metric and imperial measures. The programme calculated BMI and percentage weight loss from the weight and height data entered by patients. Patients chose the appropriate score from the BMI and percentage weight loss values displayed on the screen. The IBD-NST score was calculated by the programme.

Patients completed the electronic IBD-NST in a dedicated clinic room when attending their routine IBD out-patient appointment (record 1) and 7 days later an email was sent to patients requesting them to repeat the IBD-NST with a link that was accessible on any device to complete the electronic IBD-NST (record 2). A reminder email was sent on day 10 and day 12 ff the repeat IBD-NST had not been completed. If the repeat had not been completed by day 14 the record 1 patient data was excluded from analysis.

A test–re-test interval of 7–14 days was chosen to minimise the likelihood of changes in IBD disease activity affecting the IBD-NST score. Weight, weight loss and nutritional concerns were unlikely to vary greatly within this time period, but symptoms of a flare may have changed, especially if medication had been altered (commenced, stopped, or dose changed) during the out-patient appointment.

### 2.3. Statistical analysis

The demographics and disease-related characteristics of the patients in each cohort were not compared statistically because some patients were present in multiple cohorts, and this would violate statistical assumptions.

The content validity of all versions of the IBD-NST were compared to MUST. As part of the validation process, it was expected that the IBD-NST would identify patients at risk of malnutrition using MUST as well as patients considered to be at nutrition risk.

For convergent validity of the Draft-2 IBD-NST, the optimal combination of Step 3 questions to identify patients with impaired muscle function and at risk of malnutrition (MUST score ≥2) or malnourished (SGA-B or SGA-C) were assessed using a chi-squared test. Agreement between assessment tools and IBD-NST nutrition risk was assessed as *p* < 0.05 and this informed the step 3 components included in the final IBD-NST. The circumstances whereby patients had impaired nutritional status and were not identified by the IBD-NST were explored.

The repeat reliability of the IBD-NST was explored by comparison of record 1 test responses and record 2 re-test responses. The observed score agreement (percentage) for each question and the overall IBD-NST nutrition risk score agreement on both the test and the re-test were calculated and kappa statistic calculated for IBD-NST risk to correct for chance agreement (24).

## 3. Results

In total, 282 patients median interquartile range (IQR) age 37.3 (19.3) years and BMI 24.0 (5.4) kg/m^2^ were recruited from the outpatient clinics to develop and validate the IBD-NST (Table 1). Of these, 135 (48%) patients were male, 175 (62%) had Crohn’s disease and median (IQR) disease duration was 9.9 (15.4) years. Further demographics are reported in Table 1.

**TABLE 1:** Patient demographics and disease-related characteristics.

Characteristics	Cohort 1 (*n* = 103)	Cohort 2 (*n* = 179)	Cohort 2a (*n* = 91)	Cohort 3 (*n* = 85)	All patients (*n* = 282)^§^
Sex (male), *n* (%)	48 (47)	87 (49)	43 (47)	45 (53)	135 (48)
Age (years), median (IQR)	38.3 (20.2)	36.6 (18.9)	35.7 (20.6)	38.1 (22.0)	37.3 (19.3)
**Ethnicity, *n* (%)**					
White British	66 (64)	123 (69)	63 (69)	60 (70)	189 (67)
White other	22 (21)	24 (13)	13 (14)	14 (16)	46 (16)
Black	6 (6)	11 (6)	4 (5)	5 (6)	17 (6)
Asian	3 (3)	13 (7)	4 (5)	3 (4)	16 (6)
Other	6 (6)	8 (4)	7 (8)	3 (4)	14 (5)
**Disease, *n* (%)**					
CD	66 (64)	109 (60)	53 (58)	50 (59)	175 (62)
UC	37 (36)	55 (32)	32 (35)	27 (32)	92 (33)
IBD-U	0 (0)	15 (8)	6 (7)	8 (9)	15 (5)
Disease duration (years), median (IQR)	10.1 (15.4)	9.1 (15.6)	8.4 (13.7)	8.7 (17.4)	9.9 (15.4)
Hospitalisation in last 12 months, *n* (%)	89 (86)	80 (80)	75 (82)	64 (75)	233 (83)
No previous surgery, *n* (%)	67 (65)	104 (58)	51 (56)	54 (63)	171 (60)
**Medication, *n* (%)**					
None	23 (22)	27 (15)	15 (16)	10 (12)	50 (18)
5-aminosalicylate	28 (27)	40 (22)	28 (31)	18 (21)	68 (24)
Corticosteroid	9 (9)	10 (6)	7 (8)	3 (4)	19 (7)
Immunosuppressant	21 (20)	24 (13)	18 (20)	11 (13)	45 (16)
Biologic	18 (17)	47 (26)	17 (19)	22 (26)	65 (23)
Combination therapy*	19 (18)	50 (11)	17 (19)	30 (35)	69 (24)
Other	12 (12)	28 (16)	15 (16)	16 (19)	40 (14)
Weight (kg), median (IQR)	71.2 (22.3)	72.2 (20.0)	70.0 (19.3)	69.5 (16.8)	72.0 (20.9)
BMI (kg/m^2^), median (IQR)	23.7 (5.6)	24.2 (5.2)	23.8 (4.6)	23.6 (4.2)	24.0 (5.4)
BMI < 18.5 (kg/m^2^), *n* (%)	2 (2)	7 (4)	2 (2)	3 (4)	8 (3)
BMI 18.5–20.0 (kg/m^2^), *n* (%)	12 (11)	18 (10)	7 (8)	3 (7)	22 (8)

BMI, body mass index; CD, Crohn’s disease; IBD-U, inflammatory bowel disease unspecified; IQR, interquartile range; UC, ulcerative colitis.

^§^“All patients” is the total number of participants included in the study.

Some participants took part in more than one cohort.

*Combination therapy is use of both an immunosuppressant and a biologic and the numbers are distinct from the individual groups.

### 3.1. Face validity of IBD-NST

Draft-1 IBD-NST was completed by 103 patients in Cohort 1. Qualitative feedback on the face validity of the Draft-1 IBD-NST helped to develop Draft-2 IBD-NST (Figure 1). Patients commonly misinterpreted the MUST intended meaning of “unintentional weight loss,” “acutely sick,” and “poor oral intake.”

Patients often recorded any weight loss and either did not read the word “unintentional” or did not understand the difference been unintentional and intentional weight loss. This comment prompted reformulation of the wording for the weight loss question to ensure that patients did not score any points unless the weight loss was unplanned. The word “unintentional” weight loss was replaced with “unplanned” weight loss and an extra question was added *“Were you trying to lose weight?”* to ensure that only patients who had not planned to lose weight scored any points for this step.

Patients often questioned if MUST question *“Do you feel acutely sick right now?”* was referring to whether their IBD was active or in remission. This comment informed the development of an IBD-specific question *“Are you currently having a flare of your symptoms.”*

The MUST question “*Has your intake of food been poor for the last 5 days*…*.”* was often perceived as referring to making unhealthy food choices rather than as the intended meaning. This comment prompted the inclusion of two further questions to Step 3 *“Do you have any food and nutrition concerns”* and *“Do you currently avoid any specific foods or food groups.”*

During testing with Cohort 2, it appeared that patients (*n* = 179) understood the meaning of the new questions included in Draft-2 IBD-NST because the researchers recorded no comments from patients seeking clarification of their meaning.

### 3.2. Content validity of IBD-NST

In Cohort 1, the MUST classified 26/103 (25%) patients at moderate or high risk of malnutrition. Food and nutrition concerns were reported in 38/103 (37%) patients and another 38/103 (37%) reported they would like to see an IBD specialist dietitian if at nutrition risk. Only 2/11 (18%) patients at high risk of malnutrition on the MUST reported they would like to see an IBD specialist dietitian (Supplementary Table 1).

**FIGURE 2:**
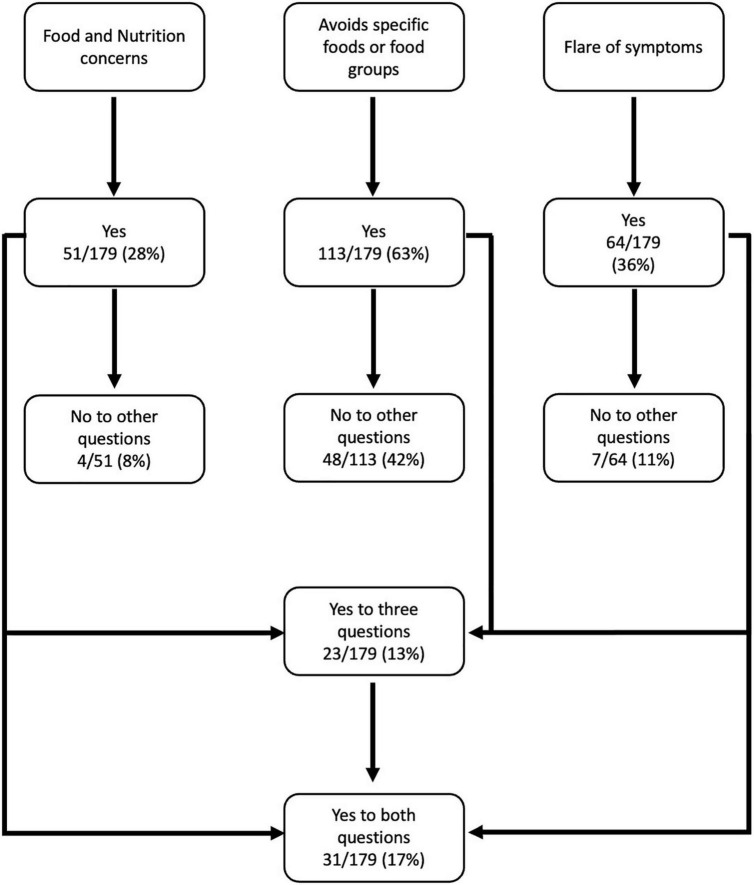
Novel IBD specific screening questions.

The content validity of Draft-2 IBD-NST was tested with Cohort 2. Step 3 contained three novel IBD-specific questions (Figure 1); 132/179 (74%) patients answered “yes” to at least one of these questions. Figure 2 shows the proportion of patients answering yes to each question and yes to a combination of these questions. Specific foods or foods groups were avoided by 113/179 (63%) of patients, however, 48/113 (42%) patients did not report food and nutrition concerns nor a flare of symptoms. Whereas, of 51/179 (28%) patients with food and nutrition concerns 31/51 (61%) patients also reported having a flare of symptoms.

In Cohort 2, 25 patients had a BMI < 20.0 kg/m^2^ of which 16/25 (64%) reported avoiding specific foods or food groups and 14/25 (56%) reported neither food and nutrition concerns nor a flare of symptoms. Only 5/25 (10%) reported both food and nutrition concerns and a flare of symptoms. This suggests that the latter questions measure a different aspect of nutrition risk compared to BMI. Similarly, 9/10 (90%) patients who reported unplanned weight loss of 5–10% body weight loss in the last 6 months avoided specific foods or foods group but 4/10 (40%) did not report food and nutrition concerns nor a flare of symptoms.

### 3.3. Convergent validity of IBD-NST

Ten patients in Cohort 2 declined to have an SGA completed so Cohort 2a comprised 91 patients (Table 2). SGA identified 10/91 (11%) patients as moderately malnourished and 1/91 (1%) patient as severely malnourished.

**TABLE 2:** Convergent validity of the Final IBD-NST and MUST compared with markers of nutritional status (Cohort 2a, *n* = 91).

		Low	Moderate	High	Total	*P*
**(a)**	**MUST risk of malnutrition**					
SGA	Well-nourished	61	11	8	80	0.001
	Mild to moderately malnourished	3	2	5	10	
	Severely malnourished	0	0	1	1	
	Total	64	13	14	91	
**(b)**	**IBD-NST nutriton risk**					
SGA	Well-nourished	58	19	3	80	<0.001
	Mild to moderately malnourished	3	5	2	10	
	Severely malnourished	0	0	1	1	
	Total	61	24	6	91	
**(c)**	**MUST risk of malnutrition**					
Muscle function	Optimal	30	2	2	34	0.015
	Impaired	34	11	12	57	
	Total	64	13	14	91	
**(d)**	**IBD-NST nutriton risk**					
Muscle function	Optimal	28	6	0	34	0.030
	Impaired	33	18	6	57	
	Total	61	24	6	91	
**(e)**	**MUST risk of malnutrition**					
Muscle mass	Optimal	39	9	8	56	0.401
	Depleted	5	2	3	10	
	Total	44	11	11	66	
**(f)**	**IBD-NST nutriton risk**					
Muscle mass	Optimal	36	15	5	56	0.9669
	Depleted	6	3	1	10	
	Total	42	18	6	66	

IBD-NST, inflammatory bowel disease nutrition self-screening tool; MUST, malnutrition universal screening tool; SGA, subjective global assessment. Muscle function was assessed using handgrip strength; impaired was defined as a maximum HGS from three attempts that was less than 85% of the mean for age and sex (16). Muscle mass was assessed using mid-upper arm muscle circumference; depleted was defined as a MAMC less than 5th centile for age and sex (17). Convergent validity was assessed using a chi-squared test.

Impaired muscle function was present in 57/91 (63%) patients. Bearing in mind that BMI is not a good marker of muscle function, impaired muscle function was observed in 6/11 (55%) patients with a BMI greater than or equal to 30 kg/m^2^ as well as, 11/12 (92%) patients with a BMI less than 20.0 kg/m^2^.

Only 66 (54%) patients agreed to have their MAMC measured, the other 25 patients declined either due to lack of time or not giving a reason. Depleted muscle mass was observed in 10/66 (15%) patients and 8/10 (80%) of these patients also had impaired muscle function.

The optimal combination of Step 3 questions of the Draft-2 IBD-NST was explored.

The proportion of patients in Cohort 2a who answered yes to each of the Step 3 questions were 35/91 (38%) to *“Are you currently having a flare of your symptoms?,”* 55/91 (60%) to *“Do you avoid specific foods or food groups?”* and 26/91 (29%) *“Do you have any food and nutrition concerns?”*

The first scoring option allocated two points if patients answered “yes” to two of the three questions. This resulted in 50/91 (55%) patients at moderate or high nutrition risk and IBD-NST score was not statistically significantly associated with impaired muscle function [χ^2^ (1, *n* = 91) = 3.71, *p* = 0.054]. The second scoring option involved: (i) Removal of the question *“Do you currently avoid any specific foods?”* from the IBD-NST because the content validity suggested that this question did not measure a unique aspect of nutrition risk. (ii) Allocated two points if patients answered “yes” to both the remaining questions (*“Do you have any food and nutrition concerns?”* and *“Are you currently having a flare of your symptoms?”)*. The second scoring option identified 30/91 (33%) of patients at moderate or high nutrition risk and there was good agreement between impaired muscle function and moderate or high nutrition risk on the IBD-NST [χ^2^ (1, *n* = 91) = 5.68, *p* = 0.017]. The inclusion of only these two questions in step 3 identified 8/11 patients malnourished on the SGA and 6/10 patients with depleted muscle mass. Three mild to moderately malnourished patients using SGA were not at nutrition risk using the IBD-NST nor at malnutrition risk using the MUST. All of these patients had a BMI between 20 and 21 kg/m^2^, but none consented to having a muscle mass assessment. One patient was 90 years old with impaired muscle function and the other two were in their early twenties having a flare of symptoms and reduced oral intake. One had had surgery (right hemicolectomy) and was on biologic medication while the other was on an immunosuppressive and biologic medication and corticosteroids.

The convergent validity of the IBD-NST was improved with the removal of the question *“Do you currently avoid any specific foods or food groups?”* therefore, the final version of IBD-NST only had two questions in Step 3 *“Are you currently having a flare of your symptoms?”* and *“Do you have any food and nutrition concerns?”* Answering yes to both questions was required to score points on step 3. Therefore, patients who score 2 points on Step 3 but 0 points elsewhere are at moderate nutrition risk.

To ensure IBD patients were correctly identified as moderate or high nutrition risk, the final IBD-NST score was compared with impaired nutritional status and risk of malnutrition measures and led to the final IBD-NST nutrition risk classifications as low (score 0), moderate (score 1 or 2) or high (score ≥3) (Table 2).

### 3.4. Repeat reliability

For Cohort 3,128 patients were approached to participate in the repeat reliability sub-study of the electronic IBD-NST. Four patients declined to participate, 124 consented and completed record 1 of electronic IBD-NST and the MUST questions and record 2 was completed by 85 (67%) patients 7–14 days later (Table 3).

**TABLE 3:** Repeat reliability of IBD-NST score.

		Record 1			
		Low	Moderate	High	Total
**Record 2**	Low	52	3	1	56 (65%)
	Moderate	4	15	3	22 (26%)
	High	0	2	5	7 (8%)
	Total	56 (65%)	20 (24%)	9 (11%)	85

IBD-NST, inflammatory bowel disease nutrition self-screening tool.

Most patients entered their weight 80/85 (94%) and height 82/85 (96%) within 95% agreement on both occasions. Five patients entered a different weight that appeared to be a data entry error, for example 25 kg the first time compared with 52 kg on the second occasion. Consequently, observed agreement for BMI score was 76/85 (89%) of patients. Unplanned weight loss was reported by 15/85 (18%), of which, five patients did not enter a similar value on both occasions. Reasons for these differences were either a patient did not report unplanned weight loss on both occasions (*n* = 1), entered very different previous weights (*n* = 2) or did not enter a previous weight (*n* = 2). There was observed agreement for unplanned weight loss score in 80/85 (94%) of patients and in 81/85 (95%) of cases for Step 3 questions “Are you currently having a flare of your symptoms” and “Do you have food and nutrition concerns.” The observed IBD-NST risk score agreement was 72/85 (85%), kappa 0.692 (95% CI 0.547 to 0.837) which shows substantial agreement.

## 4. Discussion

The IBD-NST is a novel patient-centred NST that has been validated for use in the adult IBD outpatient setting. The tool includes IBD-specific objective and subjective measures of nutritional status that better predict nutrition risk compared with tools that specifically detect malnutrition risk. The IBD-NST includes subjective questions to enable patients to evaluate their need for nutrition input and become more involved in self-management of their IBD. Patient-centred self-management approaches to IBD care have been shown to improve patient coping mechanisms, reduce symptom relapses and be cost-effective (25). The routine use of IBD-NST could facilitate timely access to dietetic care for patients during a symptom flare who are interested in dietary management approaches or for those with recent unplanned weight loss.

This NST was designed to identify patients at nutrition risk and therefore includes components that correlated with objective markers of nutritional status. Unlike MUST, which relies on BMI, weight loss and acute illness, the components of the IBD-NST comprise BMI, weight loss, IBD symptoms and nutritional concerns. A recently described IBD outpatient nutrition screening tool does not include BMI (18), however, BMI is included in the IBD-NST because low BMI is associated with more severe disease (26). BMI does not always indicate nutrition risk due to altered body composition (myopenia or sarcopenia) (27) that likely impacts upon treatment response, quality of life and risk of comorbidities (28). However, in clinical practice routine access to body composition analysis may be limited.

The current study supports that food exclusion behaviours in IBD are common (29) and demonstrated in Cohort 2 that two thirds of patients excluded at least one food. The reasons for excluding foods were not evaluated in this study but previous studies found patients believe avoiding certain foods may reduce the likelihood of an IBD flare (30, 31). A multi-variate analysis of nutrition assessment data from 333 IBD patients found that exclusion of some food groups during a disease flare was associated with risk of malnutrition, however, the exclusion of some food groups to prevent a flare was not (31). It is somewhat surprising that acute, but not chronic, dietary habits involving exclusion of food groups were associated with risk of malnutrition and suggests that the relationship between dietary intake in IBD malnutrition is complex and that perhaps an instability of food exclusions in IBD influences the association. Although other IBD nutrition screening tools include food exclusion behaviours (18), our study found that this behaviour was not associated with objective markers of nutritional status nor the presence of malnutrition and was often reported without a flare of symptoms or nutritional concerns.

A dietetic consultation, and consequently a food and nutrition intervention, that aims to improve patient symptoms is likely to have a significant impact of quality of life and/or medical interventions. In most IBD centres, there is limited dietetic resource allocated to IBD therefore the identification of patients with the most need and who are most likely to attend dietitian appointments is essential. The use of patient-centred models of care that enable patients to self-refer when they need care have been shown to reduce non-attendance rates, improve access to a responsive service without increasing service demand or waiting times and is associated with high patient satisfaction (25, 32). Furthermore, patients who are able to self-refer are more likely to attend their outpatient appointments (32, 33). A pilot study of patient-centred women’s health physiotherapy found that GP referred physiotherapy appointments were attended by 80% of patients compared to 95% of attendance for self-referred appointments (32). Similarly, patients who self-referred to psychological therapy were more likely (odds ratio of 1.04) to attend their first appointment (33). Based on previous studies in other allied health disciplines (32, 33) it appears that the implementation of a nutrition screening tool that enables self-referral could improve patient access to limited dietitian resources care while not necessarily increasing service costs.

The re-test repeatability of the IBD-NST was acceptable but could be further improved using automated electronic BMI, weight loss and IBD-NST score calculation. There was lower agreement for BMI, BMI score and IBD-NST across timespoints in part due to patient data entry errors and incorrect choice of the corresponding score. Agreement for the subjective, patient-reported and non-numerical questions was high. On a population level, self-reported weight and height provides an acceptable estimate but individual differences in recall are likely (34).

The IBD-NST is validated for use as an electronic tool which enables it to be implemented in a myriad of digital platforms. An automated calculation of BMI, weight loss and IBD-NST score reduces patient burden and the need for adequate mathematical skills or health professional calculated scores that accompany a paper based self-screening tool. The electronic IBD-NST could be included in an IBD care app, delivered with an appointment reminder *via* an electronic email link or completed on a device at the outpatient clinic when patients register at reception for their appointment. Self-completed app questionnaires are well accepted by IBD patients and have been shown to improve patient engagement in self-care management (35). The inclusion of the IBD-NST in a patient health app would enable automatic population of data such as an accurate height measurement and weight history which would further improve the test re-test reliability of the IBD-NST. Furthermore, an electronic, readily available tool could enable patients to complete the IBD-NST when they do have nutrition concerns and thus get timely access to a dietitian when they need it most. For example, to implement a nutrition treatment for active symptoms or implement a nutrition treatment plan to address significant unplanned weight loss.

The IBD-NST was validated in an outpatient setting at a large tertiary IBD referral hospital that cares for a complex patient population. The number of malnourished patients identified is lower than reported in screening studies that included both inpatients and outpatients (36) but, it is likely that the number of patients at nutrition risk in this complex validation population is higher than may be seen at other centres. The IBD-NST will identify a greater number of patients than a malnutrition screening tool. Implementation of the IBD-NST may not be a feasible option for centres with limited access to IBD specialist dietitians, however, IBD standards in the UK (37) and Australia (38) recommend the IBD multi-disciplinary team includes an IBD dietitian and patients should have access to nutritional therapies. In countries where there is limited or no access to an IBD dietitian, reducing dietetic referrals to only patients at high nutrition risk (IBD-NST ≥3) will likely identify less than 10% of the outpatient IBD population.

A limitation of the research is that the IBD-NST was specifically designed to measure IBD-related nutrition risk whereas the validation comparator tools were not. The MUST was used to screen for malnutrition and the SGA, HGS, and MAMC were used to measure impaired nutritional status.

The accuracy of how well the question “Are you having a flare of your symptoms” identified patients who were experiencing an inflammatory or functional flare of symptoms was not assessed as part of this study as patients did not complete a validated symptom tool and biomarkers of inflammation were not collected. Regardless of whether patients have inflammatory or functional symptoms they would benefit from seeing an IBD dietitian for nutritional assessment and dietary management (39).

## 5. Conclusion

The IBD-NST is a self-screening tool, validated for use as either a paper or electronic version, that identifies patients at nutrition risk who are likely to benefit from dietetic assessment and intervention. The tool enables patients with IBD-related symptoms who are concerned about their dietary intake to potentially access dietetic care to help manage their IBD. The routine use of the IBD-NST as a self-screening tool would enable patient-led care in the outpatient setting and may facilitate timely access to dietetic care.

## Data availability statement

The raw data supporting the conclusions of this article will be made available by the authors, without undue reservation.

## Ethics statement

The studies involving human participants were reviewed and approved by the North West–Liverpool East Research Ethics Committee (reference 18/NW/0062) and South Central–Oxford C Research Ethics Committee and (19/SC/0479). The patients/participants provided their written informed consent to participate in this study.

## Author contributions

CW: conceptualisation, methodology, formal analysis, investigation, and writing—original draft and reviewing and editing. BW: methodology, formal analysis, investigation, and writing—reviewing and editing. ML: conceptualisation, methodology, writing—reviewing and editing, supervision, and funding acquisition. All authors contributed to the article and approved the submitted version.
