# Supplementary material for: Development and validation of an inflammatory bowel disease nutrition self-screening tool (IBD-NST) for digital use
Source: Front Nutr. 2023 Feb 3;10:1065592. doi: 10.3389/fnut.2023.1065592 (PMC9935609; doi:10.3389/fnut.2023.1065592)
Supplement: Supplementary file 1 [file Table_1.docx]

Table S1. Comparison of MUST score with presence of food and nutrition concerns and desire to see an IBD dietitian (Cohort 1 n=103).

|  |  | **MUST** | | | |
| --- | --- | --- | --- | --- | --- |
|  |  | Low | Moderate | High | Total |
| Food & Nutrition Concerns | Yes | 26 | 6 | 6 | 38 (37%) |
|  | No | 51 | 9 | 5 | 65 (63%) |
|  | Total | 77 (75%) | 15 (15%) | 11 (11%) | 103 |
| Would like to see  an IBD dietitian | Yes, definitely | 32 | 4 | 2 | 38 (37%) |
|  | Yes, possibly | 34 | 9 | 6 | 49 (48%) |
|  | No | 11 | 2 | 3 | 16 (15%) |
|  | Total | 77 | 15 | 11 | 103 |

IBD, inflammatory bowel disease; MUST, malnutrition universal screening tool
